# Adjustment of Cell-Type Composition Minimizes Systematic Bias in Blood DNA Methylation Profiles Derived by DNA Collection Protocols

**DOI:** 10.1371/journal.pone.0147519

**Published:** 2016-01-22

**Authors:** Yuh Shiwa, Tsuyoshi Hachiya, Ryohei Furukawa, Hideki Ohmomo, Kanako Ono, Hisaaki Kudo, Jun Hata, Atsushi Hozawa, Motoki Iwasaki, Koichi Matsuda, Naoko Minegishi, Mamoru Satoh, Kozo Tanno, Taiki Yamaji, Kenji Wakai, Jiro Hitomi, Yutaka Kiyohara, Michiaki Kubo, Hideo Tanaka, Shoichiro Tsugane, Masayuki Yamamoto, Kenji Sobue, Atsushi Shimizu

**Affiliations:** 1 Division of Biobank and Data Management, Iwate Tohoku Medical Megabank Organization, Iwate Medical University Disaster Reconstruction Center, 2-1-1 Nishitokuda, Yahaba-cho, Shiwa-gun, Iwate 028–3694, Japan; 2 Division of Biomedical Information Analysis, Iwate Tohoku Medical Megabank Organization, Iwate Medical University Disaster Reconstruction Center, 2-1-1 Nishitokuda, Yahaba-cho, Shiwa-gun, Iwate 028–3694, Japan; 3 Department of Biobank, Tohoku Medical Megabank Organization, Tohoku University, 2–1 Seiryo-machi, Aoba-ku, Sendai 980–8573, Japan; 4 Department of Medicine and Clinical Science, Graduate School of Medical Sciences, Kyushu University, Maidashi 3-1-1, Higashi-ku, Fukuoka 812–8582, Japan; 5 Center for Cohort Studies, Graduate School of Medical Sciences, Kyushu University, Maidashi 3-1-1, Higashi-ku, Fukuoka 812–8582, Japan; 6 Preventive Medicine and Epidemiology, Tohoku Medical Megabank Organization, Tohoku University, 2–1 Seiryo-machi, Aoba-ku, Sendai 980–8573, Japan; 7 Epidemiology and Prevention Group, Research Center for Cancer Prevention and Screening, National Cancer Center, 5-1-1 Tsukiji, Chuo-ku, Tokyo 104–0045, Japan; 8 Laboratory of Molecular Medicine, Human Genome Center, Institute of Medical Science, The University of Tokyo, Tokyo, Japan; 9 Community Medical Supports and Health Record Informatics, Iwate Tohoku Medical Megabank Organization, Iwate Medical University Disaster Reconstruction Center, 2-1-1 Nishitokuda, Yahaba-cho, Shiwa-gun, Iwate 028–3694, Japan; 10 Division of Biomedical Information Analysis, Institute for Biomedical Science, Iwate Medical University, 2-1-1 Nishitokuda, Yahaba-cho, Shiwa-gun, Iwate 028–3694, Japan; 11 Department of Hygiene and Preventive Medicine, Iwate Medical University, 2-1-1 Nishitokuda, Yahaba-cho, Shiwa-gun, Iwate 028–3694, Japan; 12 Department of Preventive Medicine, Nagoya University Graduate School of Medicine, 65 Tsurumai-cho, Showa-ku, Nagoya 466–8550, Japan; 13 Deputy Executive Director, Iwate Tohoku Medical Megabank Organization, Disaster Reconstruction Center, Iwate Medical University, 2-1-1 Nishitokuda, Yahaba-cho, Shiwa-gun, Iwate 028–3694, Japan; 14 Department of Anatomy, School of Medicine, Iwate Medical University, 2-1-1 Nishitokuda, Yahaba-cho, Shiwa-gun, Iwate 028–3694, Japan; 15 Department of Environmental Medicine, Graduate School of Medical Sciences, Kyushu University, Maidashi 3-1-1, Higashi-ku, Fukuoka 812–8582, Japan; 16 Laboratory for Genotyping Development, Center for Genomic Medicine, RIKEN, Yokohama, Japan; 17 Division of Epidemiology and Prevention, Aichi Cancer Center Research Institute, Nagoya, Japan; 18 Department of Integrative Genomics, Tohoku Medical Megabank Organization, Tohoku University, 2–1 Seiryo-machi, Aoba-ku, Sendai 980–8573, Japan; 19 Department of Medical Biochemistry, Tohoku University Graduate School of Medicine, Seiryo-machi 2–1, Aoba-ku, Sendai 980–8575, Japan; 20 Executive Director, Iwate Tohoku Medical Megabank Organization, Disaster Reconstruction Center, Iwate Medical University, 2-1-1 Nishitokuda, Yahaba-cho, Shiwa-gun, Iwate 028–3694, Japan; 21 Department of Neuroscience, Institute for Biomedical Science, Iwate Medical University, 2-1-1 Nishitokuda, Yahaba-cho, Shiwa-gun, Iwate 028–3694, Japan; University of Kansas Medical Center, UNITED STATES

## Abstract

Differences in DNA collection protocols may be a potential confounder in epigenome-wide association studies (EWAS) using a large number of blood specimens from multiple biobanks and/or cohorts. Here we show that pre-analytical procedures involved in DNA collection can induce systematic bias in the DNA methylation profiles of blood cells that can be adjusted by cell-type composition variables. In Experiment 1, whole blood from 16 volunteers was collected to examine the effect of a 24 h storage period at 4°C on DNA methylation profiles as measured using the Infinium HumanMethylation450 BeadChip array. Our statistical analysis showed that the *P*-value distribution of more than 450,000 CpG sites was similar to the theoretical distribution (in quantile-quantile plot, λ = 1.03) when comparing two control replicates, which was remarkably deviated from the theoretical distribution (λ = 1.50) when comparing control and storage conditions. We then considered cell-type composition as a possible cause of the observed bias in DNA methylation profiles and found that the bias associated with the cold storage condition was largely decreased (λ_adjusted_ = 1.14) by taking into account a cell-type composition variable. As such, we compared four respective sample collection protocols used in large-scale Japanese biobanks or cohorts as well as two control replicates. Systematic biases in DNA methylation profiles were observed between control and three of four protocols without adjustment of cell-type composition (λ = 1.12–1.45) and no remarkable biases were seen after adjusting for cell-type composition in all four protocols (λ_adjusted_ = 1.00–1.17). These results revealed important implications for comparing DNA methylation profiles between blood specimens from different sources and may lead to discovery of disease-associated DNA methylation markers and the development of DNA methylation profile-based predictive risk models.

## Introduction

Recent epigenome-wide association studies (EWAS) have reported that the methylation level of peripheral blood DNA at hundreds of CpG sites is associated with a wide variety of diseases, including rheumatoid arthritis [1], breast cancer [2], cardiovascular diseases [3,4], and skin diseases [5], indicating that EWAS may shed light on understanding of the mechanisms of complex diseases. Although these studies have led to the successfully discovery of epigenetic markers, several confounding factors related to DNA methylation have been reported. Technical factors—such as batch effects—are a well-known artifact in DNA methylation arrays [6–8]. Biological factors include age, gender, cellular heterogeneity of blood cells, life style, and medication use [9]. Thus, controlling for potential confounders in EWAS is essential to avoid false-positive findings [9].

Large sample sizes are required in EWAS to increase a statistical power. The integration of existing biobanks can offer an adequate number of samples [10]; however, differences in DNA collection protocols among biobanks with respect to anticoagulants, time until centrifugation after blood collection, and the blood fraction used for DNA extraction (whole blood or buffy coat) may be additional potential confounders. Only few studies have examined the effect of the differences in DNA collection protocols on DNA methylation profiles. For instance, a previous study evaluated the effect of an 8 h storage period on the DNA methylation profile of blood cells (n = 4) and reported that the time-dependent variation in DNA methylation profiles was much smaller than that observed between individuals and only 0.6% of CpG sites measured by a microarray were significantly associated with storage conditions [11]; however, the systematic bias in DNA methylation profiles caused by collection procedures has yet to be fully evaluated.

In the present study, we investigated whether pre-analytical procedures involved in DNA collection could induce systematic bias in DNA methylation profiles of blood cells. In Experiment 1, we studied about systematic bias caused by a sample storage condition solely. In Experiment 2, we investigated whether differences in four DNA collection protocols used in large-scale Japanese biobanks also resulted in systematic biases. Based on these experiments, we found that pre-analytical procedures were sufficient to generate systematic biases in DNA methylation profiles and that these biases can be greatly reduced by adjusting cell-type composition changes.

## Materials and Methods

### Ethics statement

Ethical approval for the study was obtained from the Ethics Committee of Iwate Medical University (Approval ID: HG H25-1). All subjects provided written informed consent to participate in this study and provided samples anonymously.

### Experiment 1: Comparison between a cold storage condition and two control replicates

#### Blood collection and genomic DNA extraction

**Fig 1:**
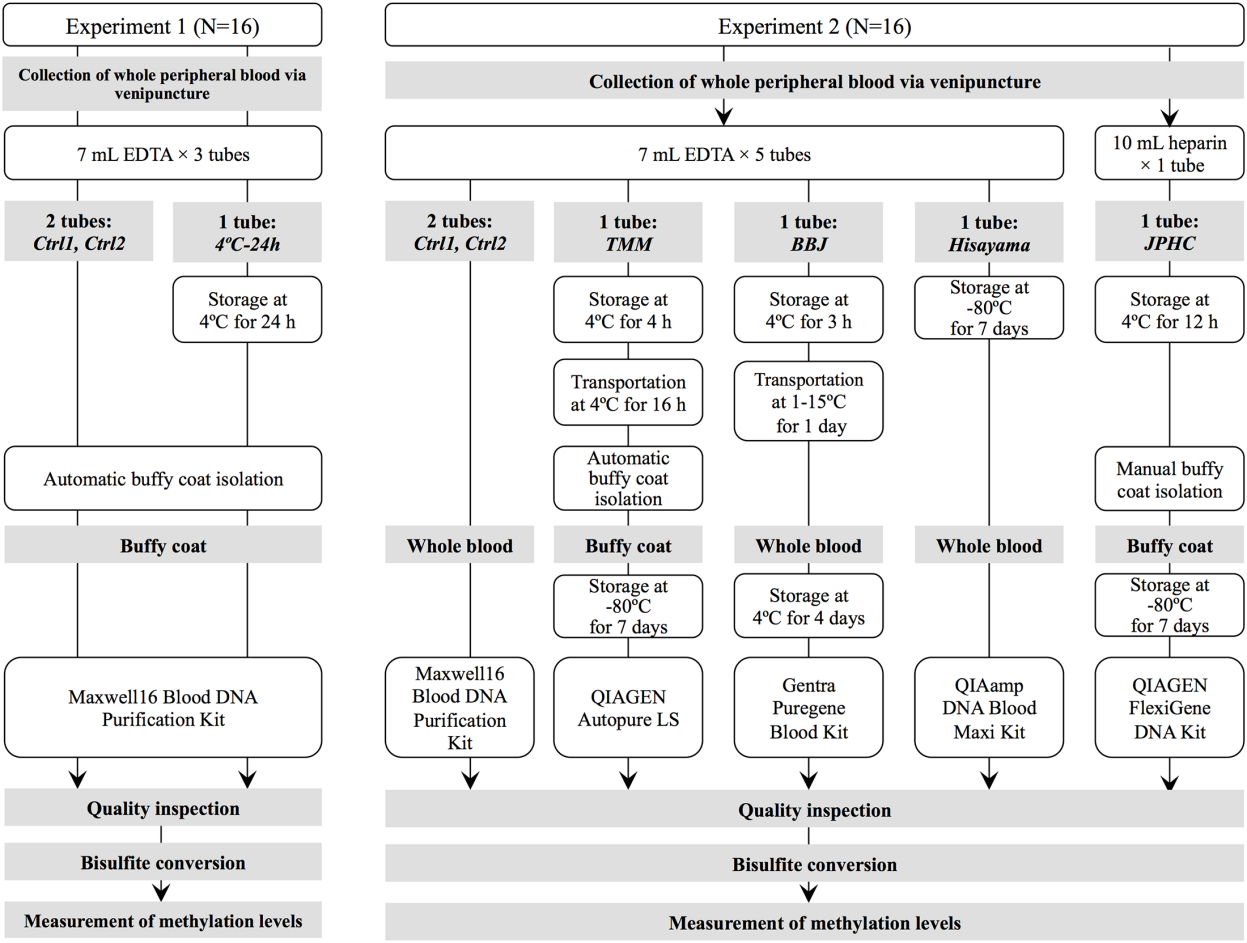
Workflow of the study design in two experiments.

The workflow is illustrated in Fig 1. Peripheral blood from 16 volunteers (10 male and 6 female) was collected in 7-mL EDTA vacutainers (Venoject II, VP-NA070K). Three tubes were collected from each volunteer, two of which were immediately processed for the isolation of buffy coats (Ctrl1 and Ctrl2 conditions). The remaining tube was stored at 4°C for 24 h (4°C-24 h condition). The tubes stored at 4°C were maintained for 30 min at room temperature prior processing. To isolate the buffy coat layer, the blood collection tubes were centrifuged at 1,500 × *g* for 10 min at room temperature using a tabletop centrifuge (Kubota Corporation, Tokyo, Japan). The buffy coat layer (700 μL) was applied to four sterile 1.3-mL tubes (FCR & Bio Co. Ltd., Kobe, Japan) automatically by a Freedom Evo100 robot (Tecan Group Ltd., Männedorf, Switzerland). Extraction of genomic DNA was performed immediately after the isolation of buffy coats. Genomic DNA was isolated using the Maxwell^®^16 Blood DNA Purification Kit on a Maxwell^®^16 Instrument according to manufacturer’s instructions. A 3-μL aliquot of extracted DNA was used for quantitative and qualitative assessment, and the remaining DNA was stored at −80°C until further use. The yield of genomic DNA was measured using the Qubit 2.0 Fluorometer (Life Technologies, Carlsbad, CA, USA) with the Qubit dsDNA BR Assay Kit. The purity of genomic DNA was assessed by the ratio of absorbance at 260 nm and 280 nm (OD_260_/OD_280_) using a Nanodrop 2000 UV-Vis Spectrophotometer (Thermo Fisher Scientific, Waltham, MA, USA). Genomic DNA integrity was evaluated by Genomic DNA ScreenTape on an Agilent 2200 TapeStation (Agilent Technologies, Santa Clara, CA, USA). All of the above procedures were performed according to manufacturer’s instructions.

#### Flow cytometry

Peripheral blood from additional 6 volunteers (1 female and 5 male) was collected and processed to isolate the buffy coats (Ctrl1, Ctrl2, and 4°C-24 h condition) as described above. The buffy coat (50 μL) was stained with 5 μL of PE/Cy7-CD3, APC-CD4, FITC-CD14, and PE-CD16 (all from Sony Biotechnology Inc., Champaign, IL, USA), and incubated for 15 min at room temperature in dark. Subsequently, 1 mL of VersaLyse lysing solution (Beckman Coulter, Brea, CA, USA) was added, immediately followed by vortexing for 1 s and incubation for 10 min at room temperature in the dark. After incubation, the sample was immediately analyzed using a Cell Sorter SH800 (Sony, Tokyo, Japan). Data from 100,000 events were collected. Lymphocyte gating was based on negative CD14 expression and low backscattered light (BSC-A); monocyte gating was based on positive CD14 expression and low BSC-A; and granulocyte gating was based on negative CD14 expression and broad BSC-A. The proportion of each subset was calculated for all events.

### Experiment 2: Comparison between four respective DNA collection protocols and two control replicates

#### Blood collection

The workflow is illustrated in Fig 1. We recapitulated four DNA collection protocols used in major biobanks and cohorts in Japan (TMM; Tohoku Medical Megabank Organization, BBJ; BioBank Japan, JPHC; Japan Public Health Center-based Prospective Study, and Hisayama; Hisayama cohort Study). We adopted the maximum duration to process the blood samples because the permissible elapsed time to process the samples differs in each protocol (S1 Table). J-MICC (Japan Multi-Institutional Collaborative Cohort Study) protocols differ with regard to the maximum time to process blood samples. Since the maximum processing time did not exceed 24 h in any of the J-MICC processing sites, we considered it suitable to substitute J-MICC protocols by the TMM protocol and exclude the former from further consideration. Since BBJ outsourced DNA extraction to three different external companies depending on the project period, we adopted the protocol used by one of the three companies. Peripheral blood samples were collected in 5 7-mL EDTA vacutainers (Venoject II, VP-NA070K, Terumo, Tokyo, Japan) and 1 10-mL heparin sodium vacutainer (Venoject II, VP-H100K, Terumo, Tokyo, Japan) from each of the 16 healthy volunteers (12 male and 4 female). Genomic DNA samples were immediately extracted from 2 of the 5 EDTA tubes (Ctrl1 and Ctrl2 protocols as the ideal conditions), and one was stored at 4°C for 4 h, followed by transportation to Tohoku University Tohoku Medical Megabank Organization at 4°C for 16 h. Next, the tubes were centrifuged, the buffy coat isolated by an automatic robot (Tecan Group, Grodig, Austria), and genomic DNA extracted (TMM protocol). Another of the ETDA tubes was stored at 4°C for 3 h and transported to BML Inc. at 1–15°C for 1 day. After that, the blood collection tube was stored at 4°C for 4 days, following the BBJ protocol of genomic DNA extraction from whole blood (BBJ protocol). The remaining EDTA tube was stored at −80°C for 7 days, and genomic DNA was extracted from whole blood (Hisayama protocol). Buffy coat was isolated from 1 heparin tube after storing the blood collection tube at 4°C for 12 h, and genomic DNA was extracted from the buffy coat (JPHC protocol).

#### Genomic DNA extraction

Fig 1 and Table 1 show the DNA extraction protocols of each biobank. For both control protocols (Ctrl1 and Ctrl2), genomic DNA was extracted from whole blood using a Maxwell16 Blood DNA Purification Kit on Maxwell16 Instrument (Promega Inc., Madison, WI, USA) according to the manufacturer’s instructions. According to the TMM protocol, the buffy coat was obtained from whole blood by centrifugation of blood collection tubes at 2,300 × *g* for 10 min at 4°C followed by automatic pipetting by a Freedom Evo robot (Tecan Group). After the buffy coat samples were stored at −80°C for 7 days, genomic DNA was automatically extracted by Autopure LS (Qiagen, Hilden, Germany). As per the BBJ protocol, genomic DNA was extracted from whole blood with the Gentra Puregene Blood Kit (Qiagen). According to the Hisayama protocol, frozen whole-blood samples were thawed at 37°C, followed by genomic DNA extraction using the QIAamp DNA Blood Maxi Kit (Qiagen). In the JPHC protocol, the buffy coat was manually obtained from centrifuged blood samples at 2,300 × *g* for 10 min at room temperature and stored at −80°C for 7 days. The genomic DNA was extracted using the FlexiGene DNA Kit (Qiagen) and was purified using Genomic DNA Clean & Concentrator-10 (Zymo Research Corporation, Orange, CA, USA). DNA quality was evaluated as described in Experiment 1.

**Table 1:** DNA collection protocols used in Experiment 2.

Protocol	Blood collection			Pre-process			DNA extraction			
	Anticoagulant	Vol. collected (mL)	Storage/transport	Centrifugation	Separation of buffy coat	Storage of buffy coat	Blood cell fraction used	DNA extraction kit (Supplier)	Vol. used	Elution volume
Control condition (Ctrl1, Ctrl2)	EDTA-2Na	7	-	-	-	-	Whole blood	Maxwell16 Blood DNA Purification Kit (Promega)	400 μL	300 μL
Tohoku Medical Megabank (TMM)	EDTA-2Na	7	4°C (16 h)	2300 × *g*, 10 min, 4°C	BC 490 μL (Automatic)	−80°C	Buffy coat	Autopure LS (Qiagen)	490 μL	350 μL
BBJ *	EDTA-2Na	7	1–15°C (1 day), 4°C (4 days)	-	-	-	Whole blood	Gentra Puregene Blood Kit (Qiagen)	7 mL	Adjust to 100 ng/μL
Hisayama	EDTA-2Na	7	−80°C (7 days)	-	-	-	Whole blood	QIAamp DNA Blood Maxi Kit (Qiagen)	7 mL	1.5 mL
JPHC	Heparin sodium	10	4°C (12 h)	10 min	BC 1–1.5 mL (Manual)	−80°C	Buffy coat	FlexiGene DNA Kit (Qiagen)	300 μL	200–500 μL

*DNA extraction outsourced to an external company.

### DNA methylation profiling using Illumina bead arrays

The Infinium HumanMethylation450 (HM450) BeadChip is an allele specific assay with more than 485,000 loci per sample, and each chip (or array) can accommodate 12 samples in a 6-row by 2-column arrangement of wells. The HM450 array has been shown to be a major source of technical biases in DNA methylation profile [6–8,12–16]. To reduce the technical bias of this array, samples derived from the same individual were loaded on the same chip (thus 4 individuals on one chip for Experiment 1, 2 individuals on one chip for Experiment 2) as displayed in S1 Fig. In Experiment 1, 48 samples (16 individuals × 3 conditions) were allocated to 4 chips as shown in S1A Fig and processed in two separate batches (S2 Table). In Experiment 2, 96 samples (16 individuals × 6 conditions) were allocated to 8 chips as shown in S1B Fig and processed in one batch (S3 Table). DNA (500 ng) was bisulfite converted with the EZ DNA methylation kit (Zymo Research Corporation) according to manufacturer’s instructions and eluted in 12 μL of elution buffer. DNA methylation profiles were measured using the Infinium HM450 BeadChip array according to the manufacturer’s instructions. In short, bisulfite-converted DNA (4 μL) was denatured, neutralized, and isothermally amplified in an Illumina hybridization oven (20–24 h). The amplified products were fragmented by an enzymatic process. After an isopropanol precipitation, the precipitated DNA was resuspended in hybridization buffer. The resuspended DNA samples were dispensed onto HM450 BeadChips (12 samples/chip). The DNA-loaded BeadChips were incubated at 48°C for 16–20 h using the Illumina hybridization oven. After this step, unhybridized DNA was washed away, and the chips were stained and subjected to single-base extension. Finally, the BeadChips were scanned using the Illumina iScan. Initial quality control was performed using Illumina GenomeStudio software (V2011.1).

### Data processing

Throughout this study, we evaluated methylation level on the basis of the β-value, which is defined as the ratio of methylated probe intensity to total signal intensity [16]. Raw intensity data (IDAT) files were imported into the R environment (v.3.1.2) using the Bioconductor minfi package (v 1.12.0) [17]. Background level correction and quantile normalization were performed using CPACOR pipeline [18]. The quality control of methylation probe was assessed by the detection *P*-value, which represents the confidence that a given probe intensity is distinguishable from a background noise [16]. We adopted a stringent detection *P* threshold of *P* < 10^−16^ in order to prevent spurious results as recommended in [18]. Only probes that passed the detection *P*-value threshold and on autosomal chromosomes were retained for further analyses. These procedures were applied to data from each of two experiments separately. A principal component analysis (PCA) and unsupervised hierarchical clustering was performed with the function prccomp and hclust in R, respectively.

### Estimation of cell-type compositions from DNA methylation profiles

To estimate cell-type compositions from DNA methylation profiles, we used the algorithm designed by Houseman et al. [19] implemented as estimateCellCounts function [20] in the minfi package with a slight modification for the compatibility with the CPACOR pipeline. DNA methylation signatures on sorted human blood cells measured by the HM450 arrays were used as a reference data set [21]. Raw intensity data files of the reference data set were processed and normalized together with those of each experiment.

### Linear regression models to test bias without adjustment of cell-type composition

We tested systematic bias between an ideal (Ctrl1) and other conditions based on linear regression analysis. In the linear regression models, principal components (PCs) of signal intensities of the control probes were included in equation terms as described in [18] to remove technical bias arising from HM450 array. To account for biological variation, we compared DNA methylation profiles and cell-type composition between conditions within the same individual. Let Δ*Y*_*ij*_ be the difference in the β-values of an individual *j* at a CpG site *i* between a condition in interest and an ideal condition (Ctrl1), $\Delta \beta_{0}^{i}$ mean difference between conditions, $\Delta X_{PC\left(k\right)}^{j}$ difference in *k*-th PCs of control probes between conditions for individual *j*, $\beta_{PC\left(k\right)}^{i}$ regression coefficients for $\Delta X_{PC\left(k\right)}^{j}$, and *ε*_*ij*_ is a residual parameter normally distributed around zero. Then, we solved the following regression model:

$$\Delta Y_{ij}=\Delta \beta_{0}^{i}+\sum_{k}\beta_{PC\left(k\right)}^{i}\times \Delta X_{PC\left(k\right)}^{j}+\varepsilon_{ij}.$$ (1)

In this model, $\Delta \beta_{0}^{i}=0$ indicates there is no difference in β-values between conditions, whereas $\Delta \beta_{0}^{i}\neq 0$ indicates some measure of difference. Accordingly, we tested whether $\Delta \beta_{0}^{i}=0$ to obtain a *P*-value for each CpG site. The *P*-value distribution of more than 450,000 CpG sites was compared to the theoretical distribution using a quantile-quantile (QQ) plot and the genomic inflation factor lambda. The genomic inflation factor lambda was calculated by dividing the median of observed chi-square statistics by the median of theoretical chi-square statistics with 1 degree of freedom [22], which quantifies the systematic bias of test statistic. In our analysis, *P* -values from linear regression were converted to chi-square statistics by the “qchisq” function in R.

### Linear regression models to test bias with adjustment of cell-type composition

To examine whether the bias in DNA methylation profiles can be corrected by adjusting cell-type composition, we added a cell-type composition variable, namely a proportion of granulocytes estimated from DNA methylation profiles (see “*Estimation of cell-type compositions from DNA methylation profiles*”, for detail), to (Eq 1) as shown in the following equation:

$$\Delta Y_{ij}\;=\;\Delta \beta_{0}^{i}+\sum_{k}\beta_{PC\left(k\right)}^{i}\times \Delta X_{PC\left(k\right)}^{j}+\beta_{Gran}^{i}\times \Delta X_{Gran}^{j}+\varepsilon_{ij}.$$ (2)

where $\Delta X_{Gran}^{j}$ represents difference in proportions of granulocytes between conditions and $\beta_{Gran}^{i}$ is a regression coefficient for $\Delta X_{Gran}^{j}$. Then, we tested whether $\Delta \beta_{0}^{i}\;=\;0$, or not, to obtain *P* values between conditions.

## Results

### Experiment 1: Comparison between a cold storage condition and two control replicates

In the first experiment, three 7 mL tubes of EDTA whole-blood samples were collected from 16 subjects (Fig 1). Two of the three tubes were immediately processed to the automatic isolation of buffy coats and DNA (Ctrl1 and Ctrl2 conditions) as duplicate controls. The remaining one tube was stored at 4°C for 24 h followed by the isolation of buffy coats and DNA (4°C-24 h condition). After DNA extraction, the yield and quality of genomic DNA were compared among the conditions. No significant difference in the yield or quality of extracted DNA was detected between conditions (S4 Table).

We measured 48 genome-wide DNA methylation profiles (16 individuals × 3 conditions) using the Illumina Infinium HumanMethylation450 array (HM450 array) in two separate batches (S2 Table). Over 470,800 good probes (≥99.35%) were detected in all samples (no samples were excluded), indicating that HM450 assays were performed with sufficiently high quality (S4 Table). To evaluate technical biases of HM450 array data, we performed an initial quality control. A similar methylation level distribution across chips (β-values) was observed (S2 Fig). A principal component analysis (PCA) showed that the samples on one chip from another batch tended to separate from other samples, indicating a non-negligible batch effect (S3A Fig). Unsupervised hierarchical clustering showed that variation of DNA methylation profiles between conditions was smaller than that between individuals (S3B Fig).

**Fig 2:**
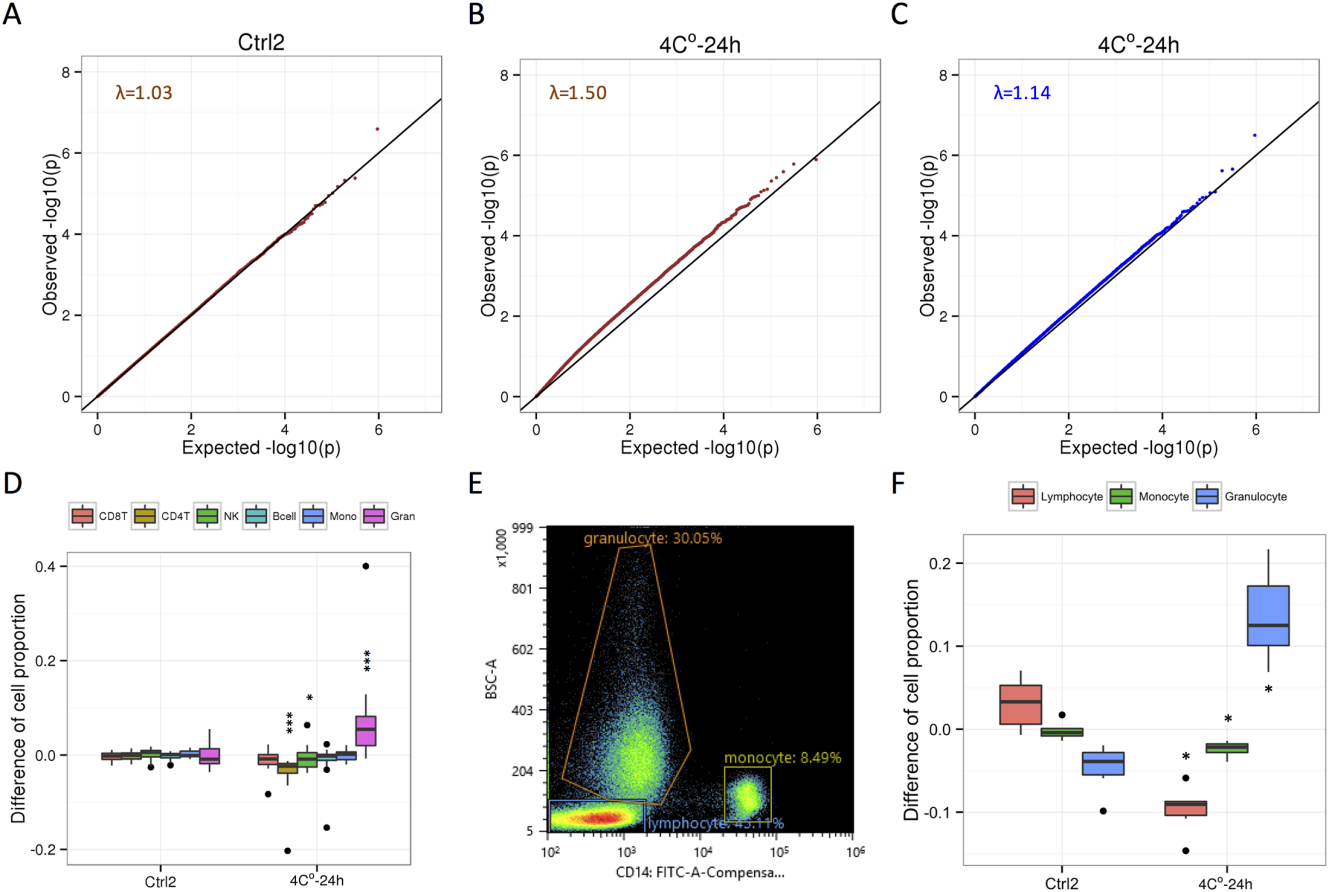
Correction of systematic biases in DNA methylation profile caused by cold storage using cell-type composition. **A**. Quantile-quantile (QQ) plot for the comparison of paired β-values from the 16 individuals between duplicates (Ctrl1 vs. Ctrl2). The genomic inflation factor lambda (median *P*-value of obs/exp) is shown. **B**. QQ plot for the comparison of 16 individuals between Ctrl1 and 4°C-24 h conditions. **C**. QQ plot for the comparison of 16 individuals between Ctrl1 and 4°C-24 h conditions after adjustment for the change in the estimated proportion of granulocytes. **D**. Differences of cell proportion between conditions (Ctrl2: Ctrl1 vs. Ctrl2; 4°C-24 h: Ctrl1 vs. 4°C-24 h) within the same individual are estimated by the cell-type composition from DNA methylation profiles. CD8T, CD8+ T cells; CD4T, CD4+ T cells; NK, natural killer cells; Bcell, B cells; Mono, monocytes; Gran, granulocytes. *, *P* < 0.05; **, *P* < 0.01; ***, *P* < 0.001 (Wilcoxon sighed rank test compared with Ctrl1) **E**. Gating strategy used to analyze populations of lymphocytes, monocytes, and granulocytes. **F**. Differences of cell proportion between conditions (Ctrl2: Ctrl1 vs. Ctrl2; 4°C-24 h: Ctrl1 vs. 4°C-24 h) within the same individual are measured by FACS using samples derived another 6 individuals. *, *P* < 0.05 (Wilcoxon signed-rank test compared with Ctrl1).

To investigate whether the 4°C-24 h condition causes biases on DNA methylation profile, we used linear regression to compare paired β-values from the 16 individuals. To reduce technical biases in HM450 array data, we applied the recently developed correction method [18], which uses principal components (PCs) of signal intensities for the HM450 array control probes (See Material and Methods). When we compared DNA methylation profiles between duplicates (Ctrl1 vs. Ctrl2) with adjustment for first 3 PCs of control probes, the *P*-value distribution of more than 450,000 CpG sites was similar to the theoretical distribution (λ = 1.03, Fig 2A and S4 Fig), indicating effectiveness of technical biases reduction of this method. When we compared DNA methylation profiles between Ctrl1 and 4°C-24 h conditions, the *P*-value distribution remarkably deviated from the theoretical distribution (λ = 1.50, Fig 2B), indicating that there was the bias caused by a cold storage.

Since whole blood is a heterogeneous collection of different cell-types, we hypothesized that the 4°C-24 h condition changes the cell-type composition of blood cells. We estimated the cell-type composition from DNA methylation profiles [19], and calculated the change of cell proportion between duplicates conditions and between Ctrl1 and 4°C-24 h conditions within the same individual (Fig 2D). No statistical difference of estimated cell-type composition between duplicates conditions (*P* > 0.05) was observed. In the 4°C-24 h condition compared with a control condition, the cell proportions of CD4T and NK were significantly decreased (*P* = 3.05 × 10^−5^ for CD4T; *P* = 2.90 ×10^−2^ for NK) and the proportion of granulocytes was significantly increased (*P* = 3.05 × 10^−5^). This observation is consistent with findings in earlier studies showing that the proportion of granulocytes in the buffy coat increased after overnight storage of whole blood at 4°C [23,24]. To confirm the change in estimated cell-type composition during cold storage, we measured the change of proportion of cell-type composition by flow cytometry using samples derived from additional six individuals (See Material and Methods). Flow cytometry analysis confirmed the tendency that the population of lymphocytes significantly decreased and the proportion of granulocytes significantly increased in in 4°C-24 h condition within the same individual (Fig 2E and 2F).

To investigate whether the change in cell-type composition could correct the systematic bias in the methylation profiles, a variable indicating the change in cell-type composition of granulocytes was added to our linear regression model as an additional covariate. As the result we found that the bias associated with the 4°C-24 h condition was largely decreased (λ = 1.14, Fig 2C). To validate our model, we randomly assign the labels of covariates using a linear regression model (S5 Fig). This could not decrease the DNA methylation biases, indicating that linear regression analysis of our models could reduce the biases caused by the 4°C-24 h condition.

### Differences in DNA collection protocols among large-scale Japanese biobanks and cohorts

To investigate whether differences in DNA collection protocols cause systematic biases on DNA methylation profile, first we surveyed DNA collection protocols among large-scale biobanks and cohorts in Japan (S1 Table). There are 3 major respects charactering the differences between the protocols, listed in S1 Table, i.e., anticoagulants (EDTA or heparin), time until centrifugation after blood collection, and the blood fraction used for DNA extraction (whole blood or buffy coat). All the biobanks and cohorts, except for JPHC, adopt an EDTA anticoagulant, whereas JPHC uses sodium heparin anticoagulant. For most biobanks, whole-blood samples are transported at a low temperature (e.g., 4°C) from collection sites to a central laboratory and are processed within 24 h. The Hisayama cohort freezes blood collection tubes at −80°C immediately after sampling.

The time from blood collection to processing and storage temperature during transportation as well as the DNA extraction method are varied between the biobanks and cohort studies (S1 Table). For example, genomic DNA is extracted from the buffy coat in the TMM protocol, while in the BBJ and Hisayama protocols, genomic DNA is extracted from whole blood. Although extraction of DNA from the buffy coat needs an additional centrifugation step, it offers not only high yields of DNA, but also other fractions such as plasma and red blood cells for future use, compared with that from whole blood. Based on protocol differences described above, we chose four DNA collection protocols (TMM, BBJ, Hisayama, and JPHC) that utilize two anticoagulants (EDTA and heparin) and different blood fractions for DNA extraction (whole blood or buffy coat).

### Experiment 2: Comparison between four DNA collection protocols and two control replicates

In Experiment 2, we employed four DNA collection protocols (TMM, BBJ, Hisayama, and JPHC) and an immediate extraction protocol (Ctrl1 and Ctrl2) as duplicate controls (Fig 1 and Table 1). After DNA extraction, the yield and quality of genomic DNA were compared among protocols (S5 Table). The mean DNA concentration derived from buffy coat DNA (TMM and JPHC) was higher than that derived from whole blood (Ctrl1, Ctrl2, BBJ, and Hisayama; *P* = 3.73 × 10^−11^). DNA quality was evaluated based on OD_260_/_280_. All DNA obtained from each condition yielded an OD_260_/_280_ of 1.8–2.0, indicating high-quality, intact DNA. Furthermore, the results of gel electrophoresis indicated that the DNA samples were of good quality and none of them had degraded (data not shown).

**Fig 3:**
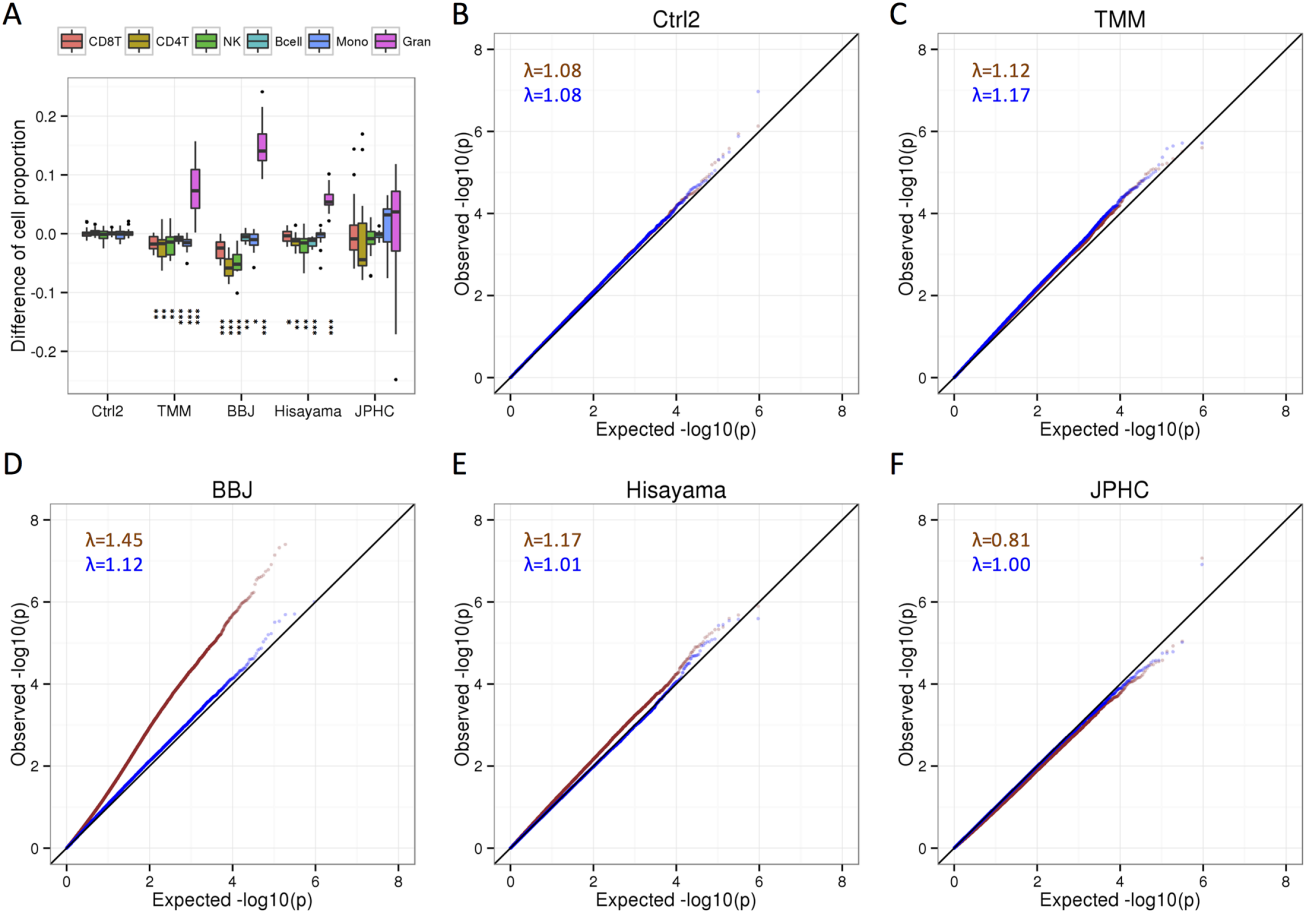
Systematic biases in DNA methylation profile caused by difference of DNA collection protocols. **A**. Differences of cell proportion between conditions (Ctrl1 vs. Ctrl2, TMM, BBJ, Hisayama, and JPHC) within the same individual are estimated by the cell-type composition from DNA methylation profiles. CD8T, CD8+ T cells; CD4T, CD4+ T cells; NK, natural killer cells; Bcell, B cells; Mono, monocytes; Gran, granulocytes. *, *P* < 0.05; **, *P* < 0.01; ***, *P* < 0.001 (Wilcoxon sighed rank test compared with Ctrl1). **B-F**. QQ plots for the comparison of 16 individuals between conditions (Ctrl1 vs. Ctrl2, TMM, BBJ, Hisayama, and JPHC) before (brown points) and after adjustment for the change in the estimated proportion of granulocytes (blue points). The genomic inflation factor lambda (median *P*-value of obs/exp) is shown.

We measured 96 genome-wide DNA methylation profiles (16 individuals × 6 conditions) using the HM450 array in one batch (S3 Table) and evaluated the suitability of these DNA preparations for HM 450 array analysis. Over 472,351 good probes (≥99.68%) were detected in all samples (no samples were excluded), indicating that all DNA collection protocols evaluated in this study retained sufficient genomic DNA quality for DNA methylation profiles (S5 Table). Similar β-values were observed across chips (S6 Fig). PCA showed that the samples on one chip tended to separate from other samples (S7 Fig). Unsupervised hierarchical clustering showed that variation of DNA methylation profiles between conditions was smaller than that between individuals (S8 Fig). Two samples (CY5 and DC5) processed by JPHC protocol were not included in each individual cluster, possibly due to manual isolation of buff coats, which may lead to a large variation of cell-type compositions. Estimated cell-compositions were significantly different between a control condition and TMM, Hisayama and BBJ protocols (Fig 3A). For the JPHC protocol, the difference was not significant, whereas the estimated cell-compositions had large variations, possibly due to the manual isolation in the protocol.

To investigate whether differences in the DNA collection protocols resulted in systematic biases on DNA methylation profile, we compared paired β-values from the 16 individuals using a linear regression. When we compared duplicated control samples (Ctrl1 vs. Ctrl2) with adjustment for first 3 PCs of control probes, the *P*-value distribution of more than 450,000 CpG sites is similar to the theoretical distribution (λ = 1.08 Fig 3B and S4 Fig). When we compared DNA methylation profiles between Ctrl1 and each protocol, the *P*-value distribution was remarkably deviated from the theoretical distribution in the BBJ protocol (λ = 1.45; Fig 3D). For the Hisayama protocol, λ was slightly inflated (λ = 1.17; Fig 3E). The *P*-value distribution in the TMM protocol was similar to the theoretical distribution (λ = 1.12, Fig 3C). For the JPHC protocol, λ = 0.81, implying that large variations of cell-type composition may decrease the power of tests (Fig 3F). In Hisayama protocols, and remarkably deviated from the theoretical distribution in BBJ, indicating systematic biases in DNA methylation profiles between control and the three protocols.

After the adjustment by a cell-type composition variable indicating the difference of granulocyte proportions, the *P*-value distributions of the four protocols were similar to the theoretical distribution (λ_adjusted_ ranged from 1.00 to 1.17; Fig 3), whereas the *P*-value distributions were not similar in permutation tests (S9 Fig). For the BBJ and Hisayama protocols, wherein the systematic bias was the largest and the second largest, respectively, the bias was substantially decreased. For the TMM protocol, λ did not decrease because the bias was not evident before the adjustment. Interestingly, the λ was close to 1.0 after the adjustment for the JPHC protocol (Fig 3F), indicating that the power of tests may be improved by the adjustment of the cell-type compositions when the manual isolation is included in sample collection protocols.

## Discussion

To our knowledge, this is the first finding that differences in DNA collection protocols cause systematic bias in DNA methylation profiles. Hebels et al. [11] reported that condition-dependent variation in DNA methylation profiles was much smaller than that observed between individuals, which is consistent with our experimental data (S3B Fig). This result indicated that a careful analytical method would be necessary to compare DNA methylation profiles derived from the same individuals with varying conditions and address the systematic bias caused by these differences. For that purpose, we made use of the quantile-quantile plot, which compares observed and theoretical *P* value distributions and is suited for the detection of systematic bias. This methodology revealed that pre-analytical procedures were sufficient to induce systematic bias in DNA methylation profiles.

Through these experiments, we showed that changes of cell-type composition due to pre-analytical procedures are a major source of bias. The cause of the change of cell-type compositions is different due to blood cell fraction used for DNA extraction. For buffy coat, previous studies demonstrated that buffy coat separated after overnight storage of whole blood at 4°C was contaminated with a large number of granulocytes due to the change of the specific density of granulocytes [23,24]. For DNA derived from whole-blood, previous study showed that the number of lymphocytes (CD4T and CD8T) was found to decrease after frozen storage of whole blood at −80°C [25]. It is possible that by BBJ and Hisayama protocols, decreasing lymphocyte cells due to storage causes an apparent increase in the granulocyte proportion (Fig 3A). Although the adjustment of cell-type composition has been used in previous EWAS [1,20,26,27], this is the first report showing that the bias in DNA methylation profiles intrinsic to DNA collection protocols can be corrected by adjustment of cell-type composition variables.

While we showed that the adjustment of cell-type composition variables could be corrected confounding by DNA collection protocols, other sources of confounding factors must be considered. Batch effects are major technical confounders related to array experimental factors such as experimental day and chip position [7]. To avoid the batch effects across chips, samples derived from the same individual were loaded on the same chip as displayed in S1 Fig. Since each condition was assigned to the specific row position of chip, there is a concern of confounding between chip position and conditions. When we compared DNA methylation profiles between control duplicates in two experiments, we observed the *P*-value distribution slightly deviated from the theoretical distribution (λ = 1.28, S4 Fig), indicating that there was the technical bias caused by chip position. To address these issues, we employed recently developed CPACOR pipeline, which uses principal components (PCs) of signal intensities of the control probes for statistical adjustments [18]. This method successfully removed technical bias between duplicate conditions (S4 Fig). Although we cannot completely exclude the possibility of confounding between chip position and conditions, it is worth noting that our statistical adjustment models could reduce the bias in DNA methylation profiles intrinsic to DNA collection protocols using cell-type composition variables.

In conclusion, we found that pre-analytical procedures cause systematic biases in DNA methylation profiles and the biases are greatly reduced by adjusting cell-type composition changes. Our results provided important implications for comparing DNA methylation profiles between blood specimens from different sources and will lead to discovery of DNA methylation markers associated with diseases as well as to the development of DNA methylation profile-based predictive risk models.

## Supporting Information

S1 Fig Chip layout in Experiment 1 (A) and Experiment 2 (B). **A**. Three conditions (Ctrl1, Ctrl2, and 4°C-24 h) are assigned to the specific row positions. **B**. Six conditions (Ctrl1, Ctrl2, TMM protocol, BBJ protocol, JPHC protocol, and Hisayama protocol) are assigned to the specific row position. Samples derived from the same individual are assigned to the same column (C01 or C02), and highlighted by the same color.
(PDF)

S2 Fig DNA methylation for 48 samples presented as boxplots in Experiment 1. Box plot of normalized beta values for four chips of 48 samples. Each color represents a distinct chip. One chip (purple) is the different batch.
(PDF)

S3 Fig Relationship of samples based on DNA methylation profiles in Experiment 1. **A**. PCA plot for 48 samples. Each color represents a distinct chip. One chip (purple) is the different batch. **B**. Unsupervised hierarchical clustering for 48 samples. Samples from the same individual (A-P) are labeled with the initial letter. Duplicates (Ctrl1 and Ctrl2) and 4°C-24 h conditions from individual are labeled with 1, 2, and 4, respectively. Red bars indicate samples derived from the same individual are clustered together.
(PDF)

S4 Fig Correction of technical biases between duplicates using control probes in two experiments. Lehne et al. developed a new method to correct for technical biases in the HM450 array data using PCs (PC1-3) of intensities of control probes [18]. In two experiments (Ex1: Experiment 1; and Ex2: Experiment 2), we compared QQ plots for the comparison of 16 individuals between duplicates (Ctrl1 vs. Ctrl2) with no adjustments, first 1 PC (PC1), two PCs (PC1-2), and three PCs (PC1-3) of control probes.
(PDF)

S5 Fig QQ plot adjustment for shuffled covariates in Experiment 1. QQ plot for the comparison of 16 individuals between Ctrl1 and 4°C-24 h conditions after adjustment for the value of shuffled covariates (brown points: PCs of control probes as covariates; blue points: additional covariates of the change in the estimated proportion of granulocytes).
(PDF)

S6 Fig DNA methylation for 96 samples presented as boxplots in Experiment 2. Box plot of normalized beta values for eight chips of 96 samples. Each color represents a distinct chip. All chips are the same batch.
(PDF)

S7 Fig PCA plot for 96 samples in Experiment 2. Each color represents a distinct chip. Although all chips are the same batch, the samples on one chip (orange) tended to separate from other samples.
(PDF)

S8 Fig Unsupervised hierarchical clustering for 96 samples. Samples from the same individual (CL-DD) are labeled with the initial letter. Six conditions from individual are labeled with 1: Ctrl1; 2: Ctrl2; 3: TMM; 4: BBJ; 5: JPHC; 6: Hisayama, respectively. Red bars indicate samples derived from the same individual are clustered together. Blue bar indicates samples are clustered separately from each individual cluster.
(PDF)

S9 Fig QQ plots adjustment for shuffled values in Experiment 2. QQ plot for the comparison of 16 samples between conditions (Ctrl1 vs. Ctrl2, TMM, BBJ, Hisayama, and JPHC) after adjustment for the shuffled value of three PCs (PC1-3) of control probes (brown points) and in addition to the change of cell proportion of granulocytes (blue points).
(PDF)

S1 Table DNA collection protocols among major cohorts and biobanks in Japan. (XLSX)

S2 Table Sample allocation of HM450 array in Experiment 1. (XLSX)

S3 Table Sample allocation of HM450 array in Experiment 2. (XLSX)

S4 Table Purity and quantity of DNA and quality of beads arrays in Experiment 1. (XLSX)

S5 Table Purity and quantity of DNA and quality of beads arrays in Experiment 2. (XLSX)
