# Supplementary material for: Adjustment of Cell-Type Composition Minimizes Systematic Bias in Blood DNA Methylation Profiles Derived by DNA Collection Protocols
Source: PLoS One. 2016 Jan 22;11(1):e0147519. doi: 10.1371/journal.pone.0147519 (PMC4723336; doi:10.1371/journal.pone.0147519)
Supplement: S1 Fig — A. Three conditions (Ctrl1, Ctrl2, and 4°C-24 h) are assigned to the specific row positions. B. Six conditions (Ctrl1, Ctrl2, TMM protocol, BBJ protocol, JPHC protocol, and Hisayama protocol) are assigned to the specific row position. Samples derived from the same individual are assigned to the same column (C01 or C02), and highlighted by the same color. (PDF) [file pone.0147519.s001.pdf]

A: Experiment 1

	C01	C02
R01	Ctrl1	Ctrl1
R02	Ctrl2	Ctrl2
R03	4°C-24h	4°C-24h
R04	Ctrl1	Ctrl1
R05	Ctrl2	Ctrl2
R06	4°C-24h	4°C-24h

B: Experiment 2

	C01	C02
R01	Ctrl1	Ctrl1
R02	Ctrl2	Ctrl2
R03	TMM	TMM
R04	BBJ	BBJ
R05	JPHC	JPHC
R06	Hisayama	Hisayama

S1 Fig.
