# Supplementary figures and images for: Adjustment of Cell-Type Composition Minimizes Systematic Bias in Blood DNA Methylation Profiles Derived by DNA Collection Protocols
Source: PLoS One. 2016 Jan 22;11(1):e0147519. doi: 10.1371/journal.pone.0147519 (PMC4723336; doi:10.1371/journal.pone.0147519)

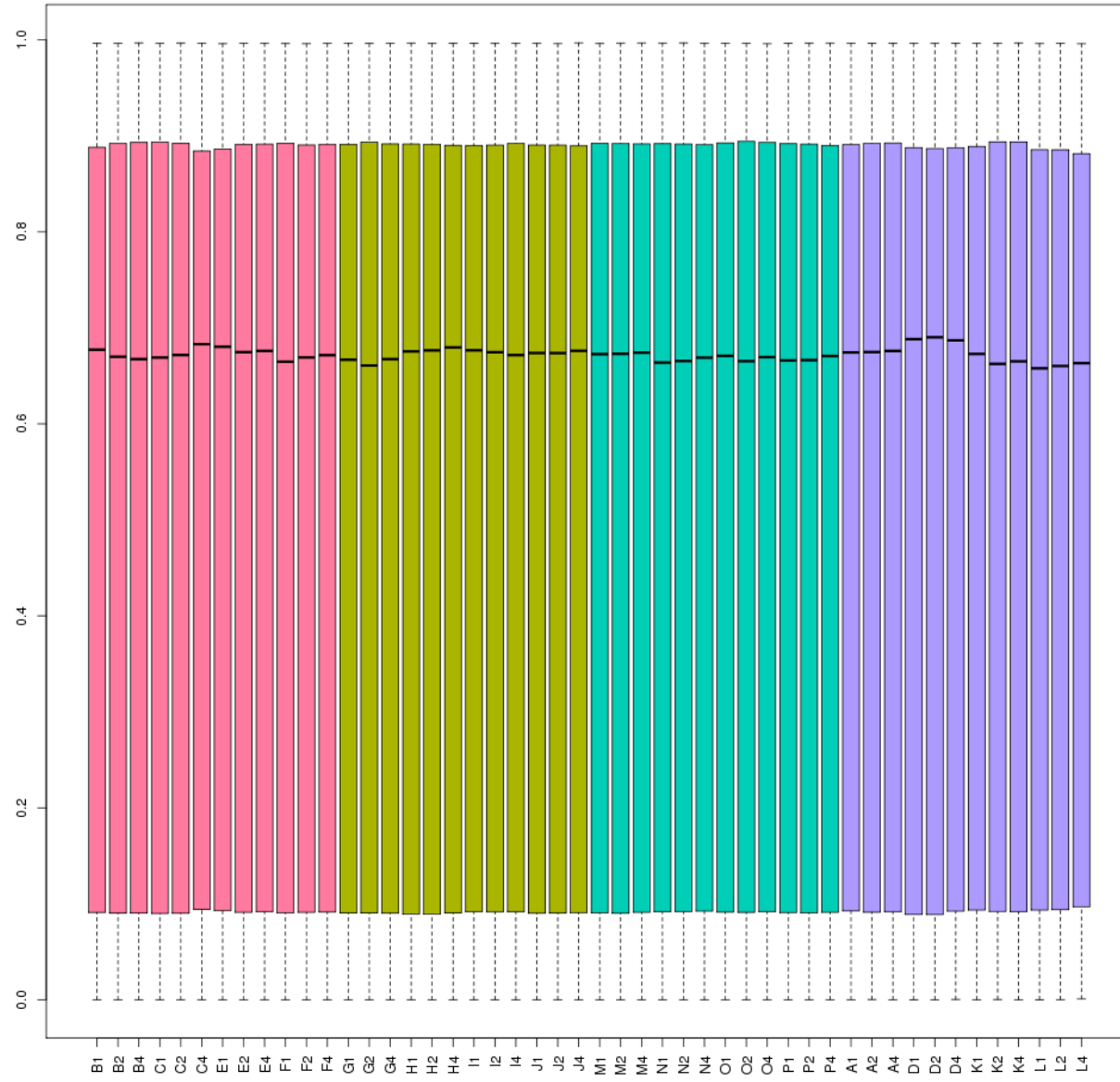

S2 Fig.

Supplement: S2 Fig — Box plot of normalized beta values for four chips of 48 samples. Each color represents a distinct chip. One chip (purple) is the different batch. (PDF) [file pone.0147519.s002.pdf]

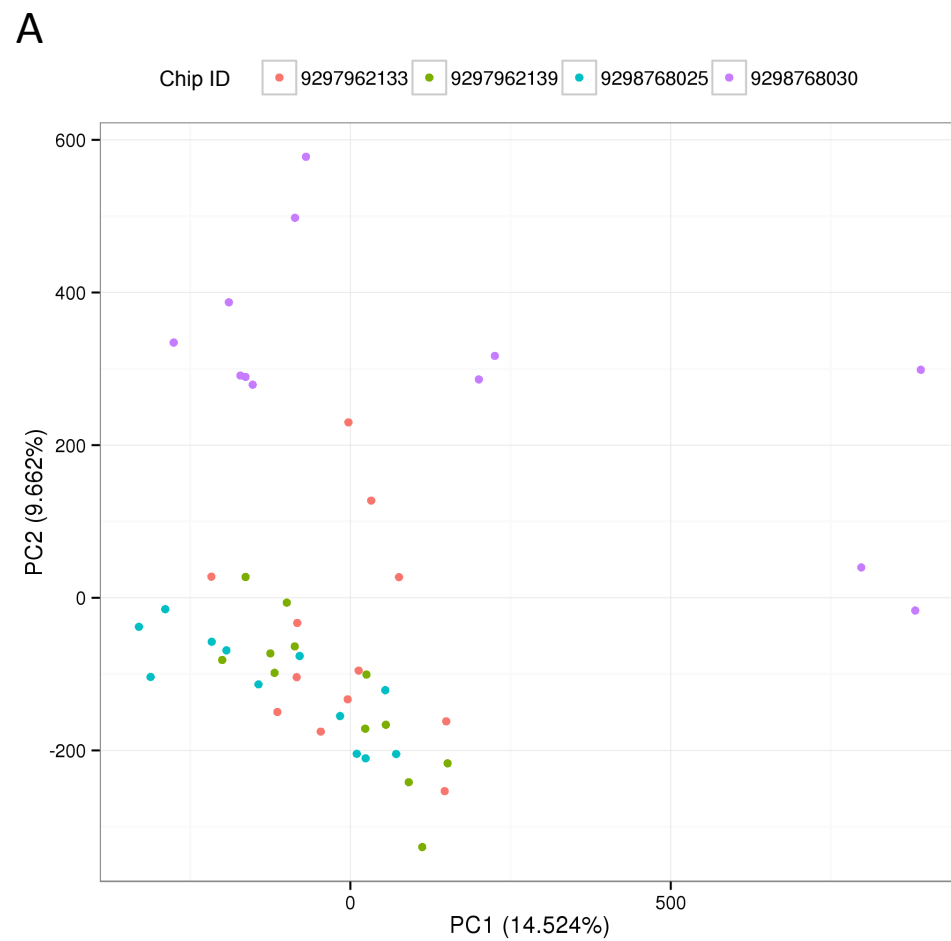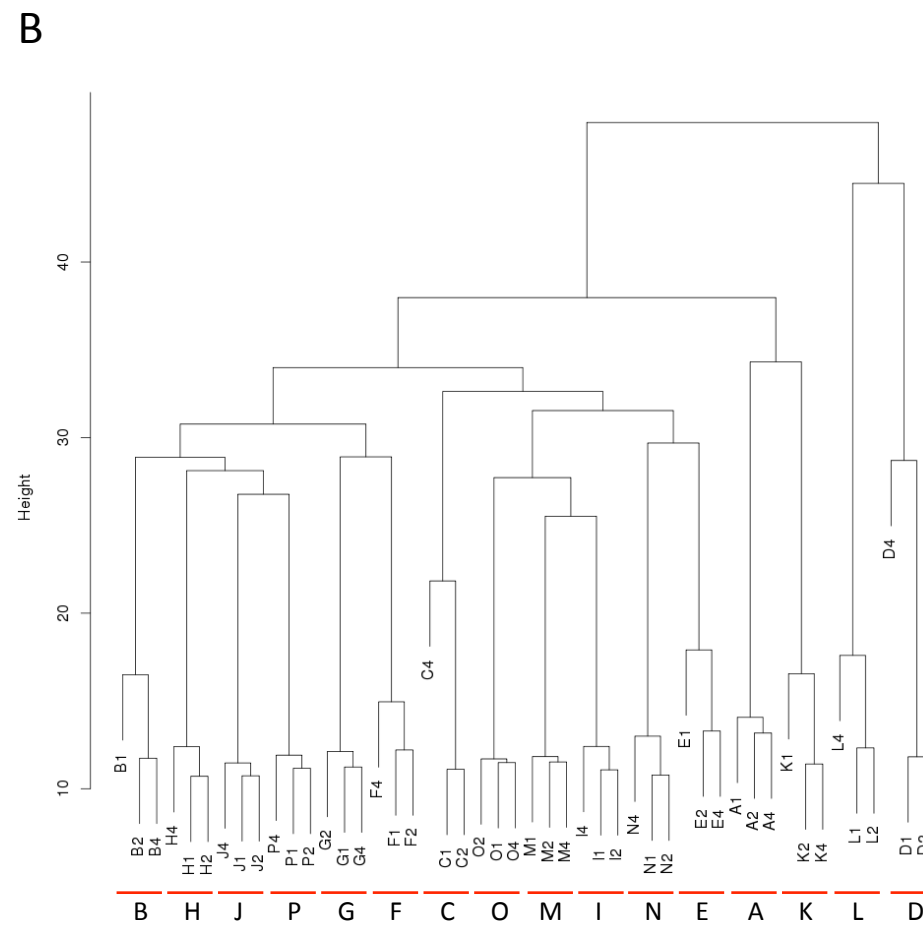

S3 Fig.

Supplement: S3 Fig — A. PCA plot for 48 samples. Each color represents a distinct chip. One chip (purple) is the different batch. B. Unsupervised hierarchical clustering for 48 samples. Samples from the same individual (A-P) are labeled with the initial letter. Duplicates (Ctrl1 and Ctrl2) and 4°C-24 h conditions from individual are labeled with 1, 2, and 4, respectively. Red bars indicate samples derived from the same individual are clustered together. (PDF) [file pone.0147519.s003.pdf]

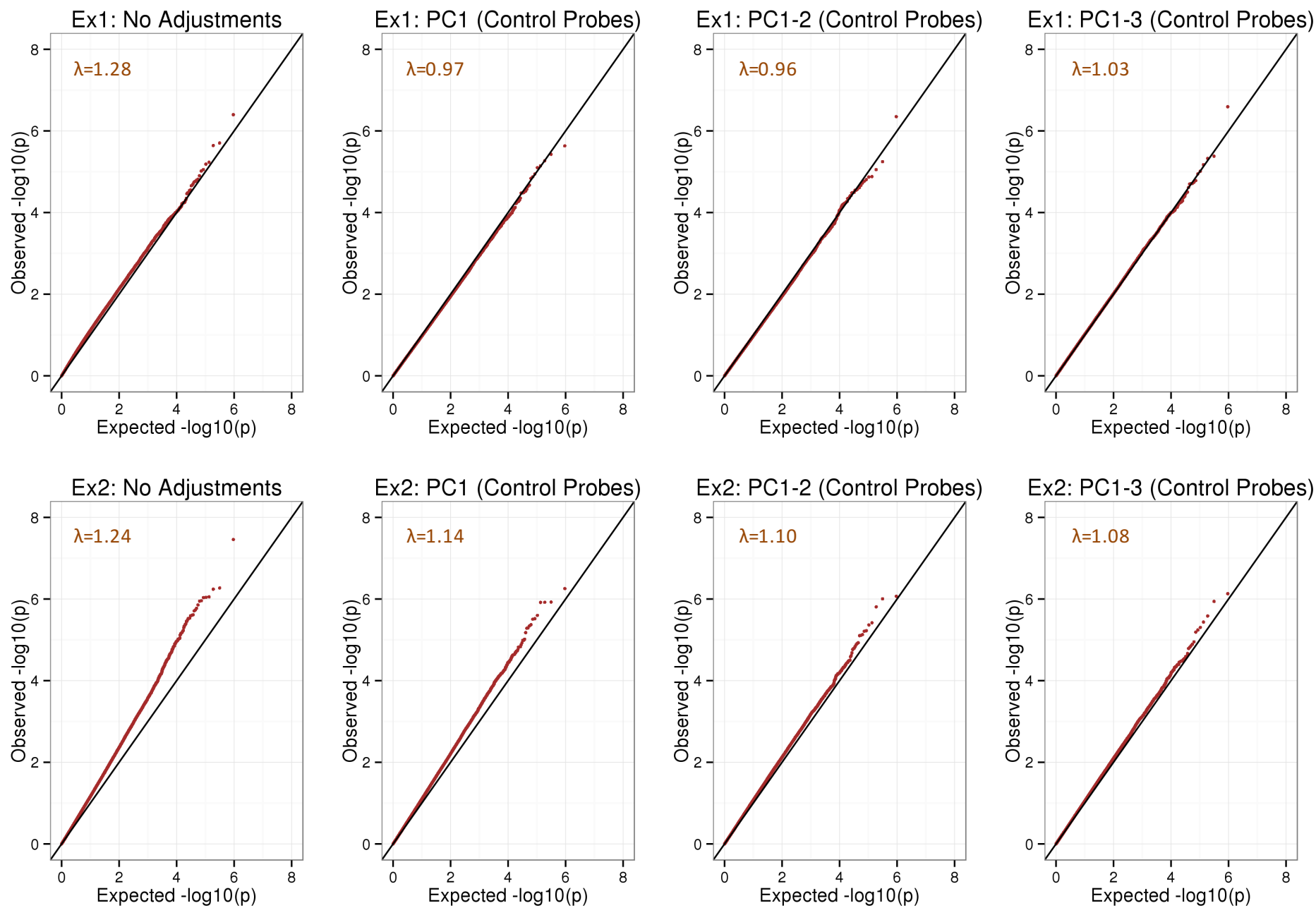

S4 Fig.

Supplement: S4 Fig — Lehne et al. developed a new method to correct for technical biases in the HM450 array data using PCs (PC1-3) of intensities of control probes [18]. In two experiments (Ex1: Experiment 1; and Ex2: Experiment 2), we compared QQ plots for the comparison of 16 individuals between duplicates (Ctrl1 vs. Ctrl2) with no adjustments, first 1 PC (PC1), two PCs (PC1-2), and three PCs (PC1-3) of control probes. (PDF) [file pone.0147519.s004.pdf]

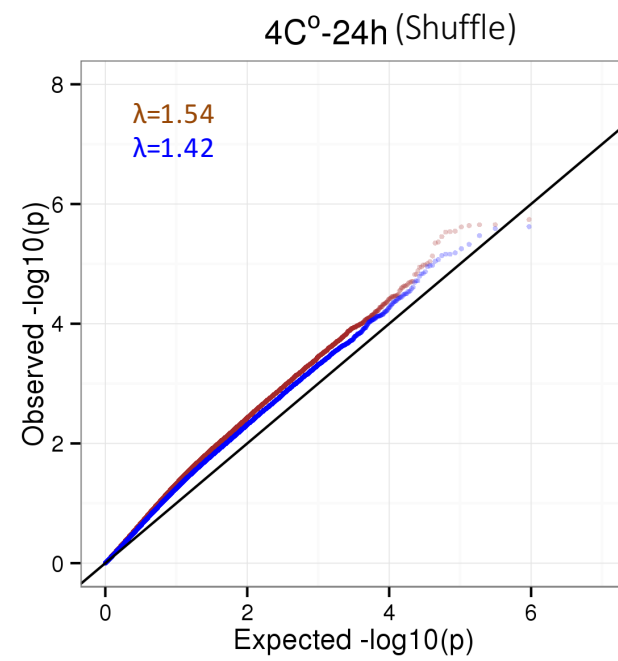

S5 Fig.

Supplement: S5 Fig — QQ plot for the comparison of 16 individuals between Ctrl1 and 4°C-24 h conditions after adjustment for the value of shuffled covariates (brown points: PCs of control probes as covariates; blue points: additional covariates of the change in the estimated proportion of granulocytes). (PDF) [file pone.0147519.s005.pdf]

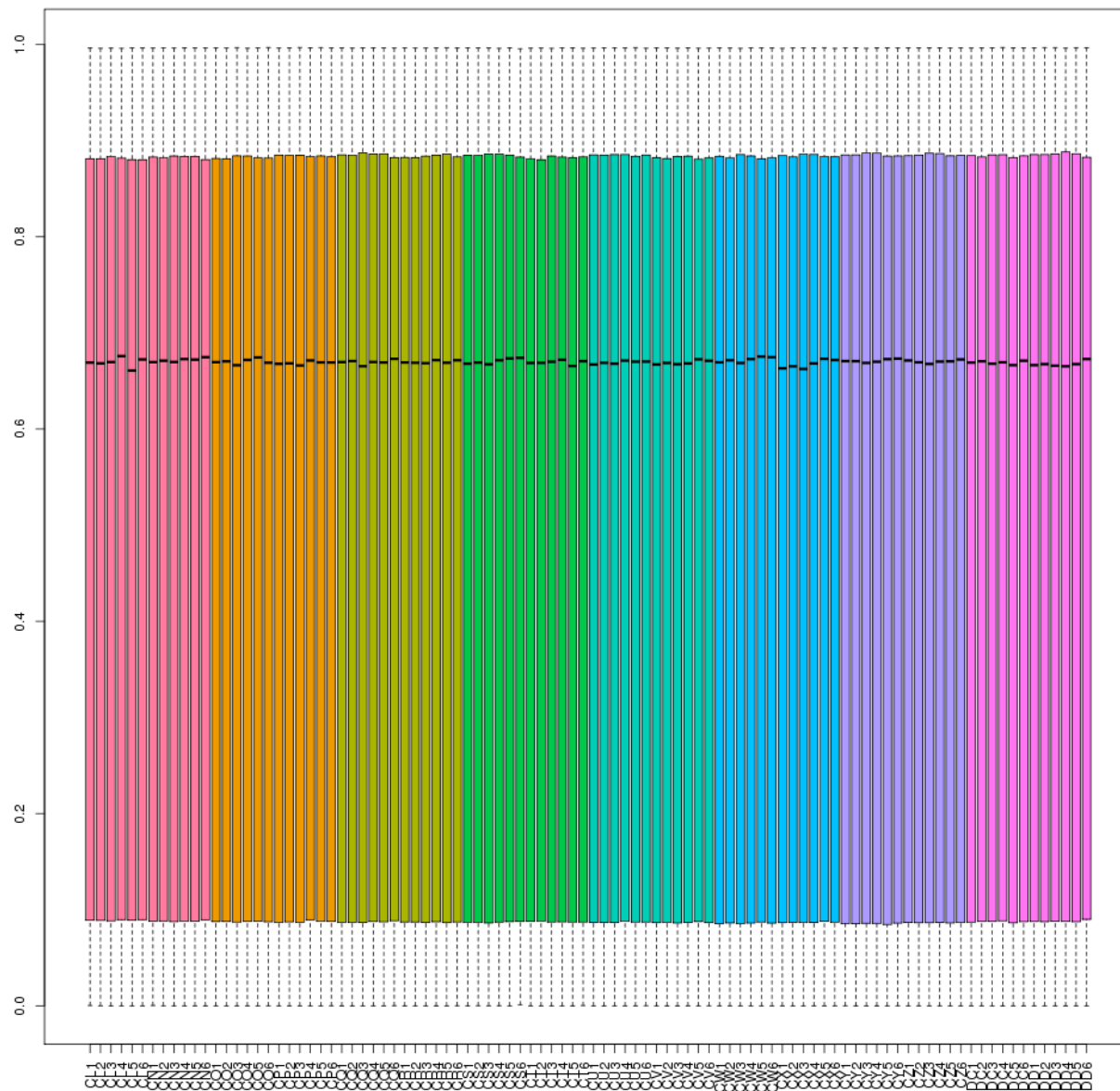

S6 Fig.

Supplement: S6 Fig — Box plot of normalized beta values for eight chips of 96 samples. Each color represents a distinct chip. All chips are the same batch. (PDF) [file pone.0147519.s006.pdf]

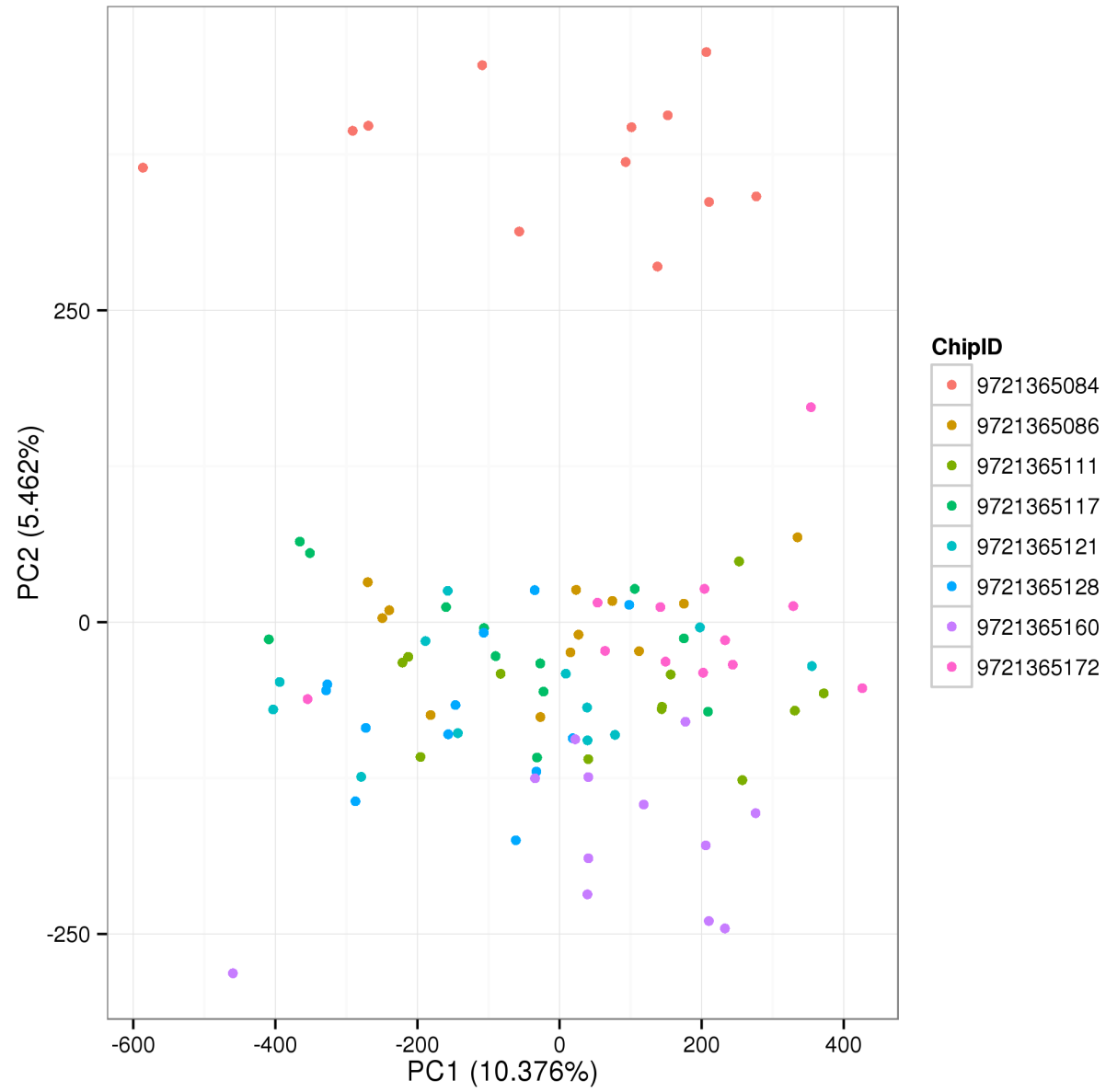

S7 Fig.

Supplement: S7 Fig — Each color represents a distinct chip. Although all chips are the same batch, the samples on one chip (orange) tended to separate from other samples. (PDF) [file pone.0147519.s007.pdf]

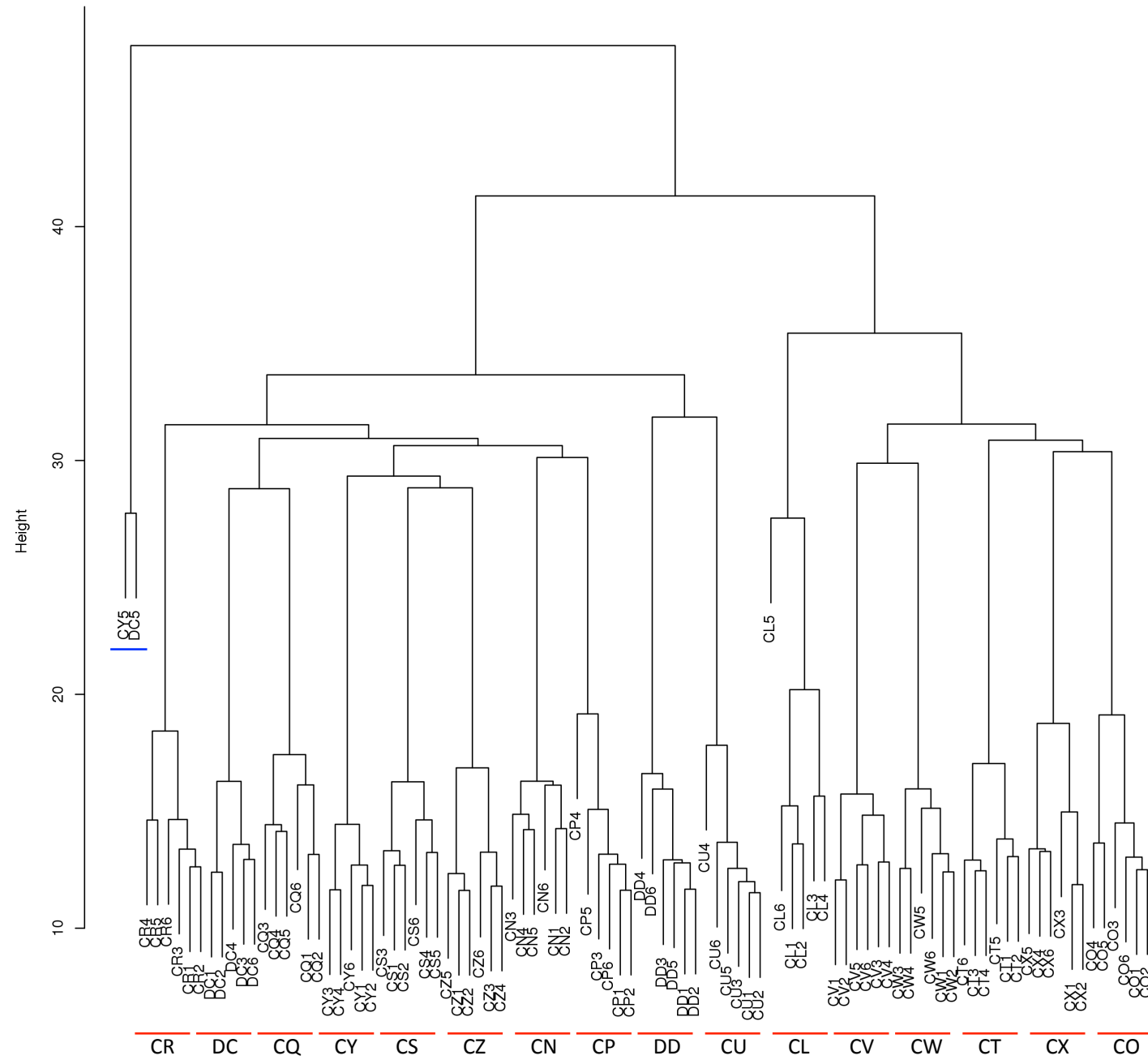

S8 Fig.

Supplement: S8 Fig — Samples from the same individual (CL-DD) are labeled with the initial letter. Six conditions from individual are labeled with 1: Ctrl1; 2: Ctrl2; 3: TMM; 4: BBJ; 5: JPHC; 6: Hisayama, respectively. Red bars indicate samples derived from the same individual are clustered together. Blue bar indicates samples are clustered separately from each individual cluster. (PDF) [file pone.0147519.s008.pdf]

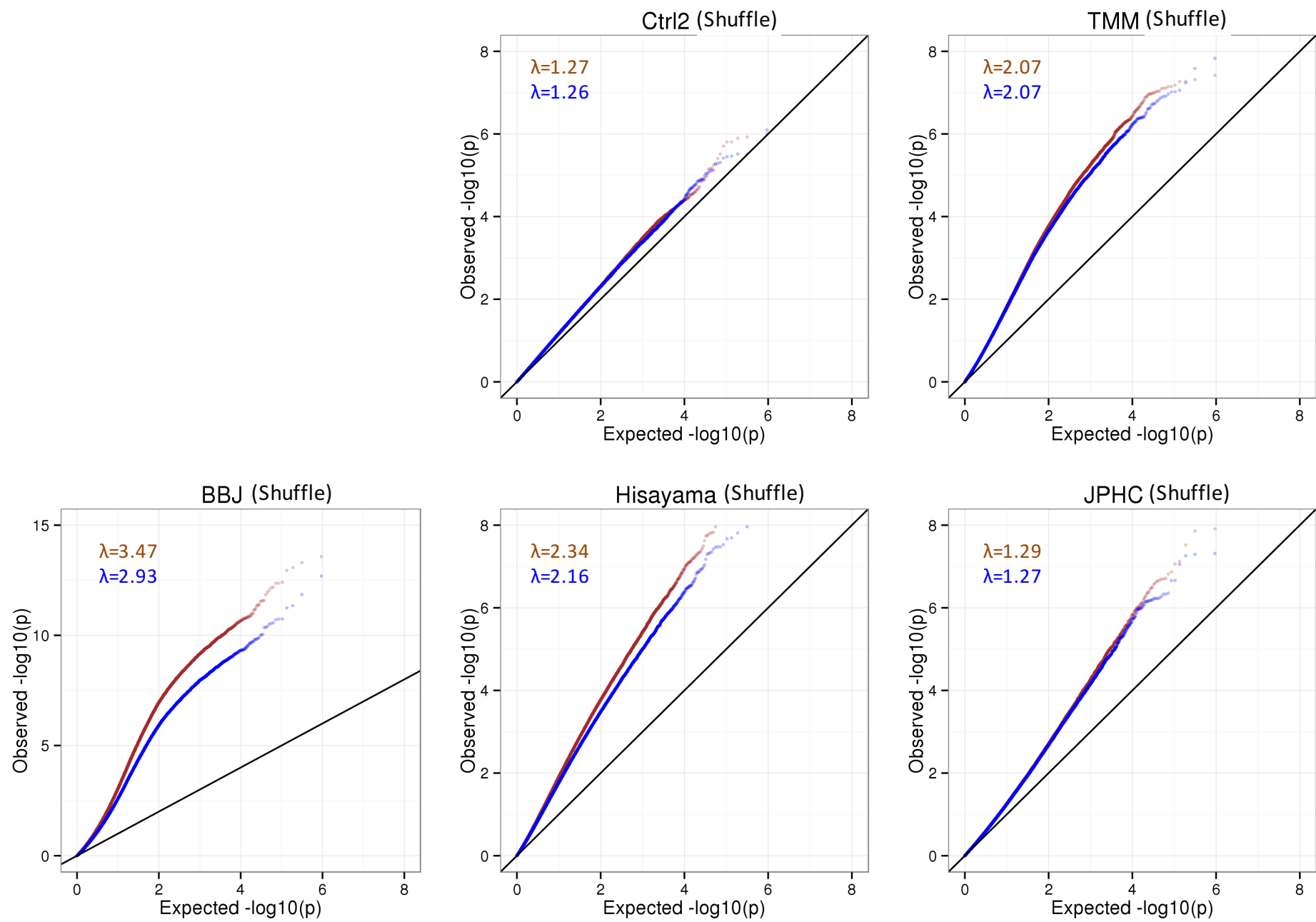

S9 Fig.

Supplement: S9 Fig — QQ plot for the comparison of 16 samples between conditions (Ctrl1 vs. Ctrl2, TMM, BBJ, Hisayama, and JPHC) after adjustment for the shuffled value of three PCs (PC1-3) of control probes (brown points) and in addition to the change of cell proportion of granulocytes (blue points). (PDF) [file pone.0147519.s009.pdf]
